# A bibliometric analysis of the 100 top-cited systematic review and meta-analysis in Orthodontics

**DOI:** 10.1590/2177-6709.29.2.e242401.oar

**Published:** 2024-06-10

**Authors:** Madhanraj SELVARAJ, Bhaskar NIVETHITHA, Piramanayagam VARSHITHA, Ulaganathan SANGEETHA, Balasubramanian MADHAN

**Affiliations:** 1 Jawaharlal Institute of Postgraduate Medical Education and Research (JIPMER), Department of Dentistry, Division of Orthodontics and Dentofacial Orthopedics (Puducherry, India).

**Keywords:** Bibliometrics, Citation analysis, Orthodontics, Systematic reviews, Meta-analysis, Bibliometria, Análise de citações, Ortodontia, Revisões sistemáticas, Meta-análise

## Abstract

**Objective:**

This bibliometric study aimed to analyze the citation metrics, journal and author characteristics, and subject domains of the 100 top-cited Systematic Reviews (SR) and Meta-Analysis (MA) in orthodontics.

**Material and Methods:**

An electronic database search was conducted for SR and MA in the Web of Science on 16^th^ July 2023, without language and time restrictions. Of the 802 hits returned, the 100 top-cited orthodontic articles were shortlisted. They were analyzed for citation metrics, journal characteristics (journal, year of publication, impact factor-IF), author and affiliation characteristics (number, primary and corresponding author’s affiliation, and country), study domain, and keywords.

**Results:**

These articles were published from 1996 to 2021 in 20 journals, with an impact factor of 1.9 to 10.5, by 351 researchers affiliated with 104 universities. Their citations ranged from 45 to 344, and 34 poised to be classified as classic (≥ 100 citations). The maximum number of articles was published in the American Journal of Orthodontics and Dentofacial Orthopedics (n=38), the European Journal of Orthodontics (n=18), and the Angle Orthodontist (n=8). The authors for individual papers ranged from 1 to 10, with 5 being the most common (n=58). Europe had the highest contribution regarding the number of corresponding authors, institutions, and citations. Bone anchorage and orthodontic tooth movement/Biomechanics were the most frequently researched domains (n=11 each). The most common keyword used was Orthodontics (n=19), followed by Systematic Review (n=16) and Meta-analysis (n=9).

**Conclusion:**

In general, the top cited SR and MA were published in high-impact orthodontic journals, were multi-authored, and reflected the collaborative work from different universities.

## RESUMO

**Objetivo:**

Este estudo bibliométrico teve como objetivo analisar as métricas de citação, as características dos periódicos e dos autores, e os domínios temáticos das 100 Revisões Sistemáticas (RS) e Meta-Análises (MA) mais citadas em Ortodontia.

**Material e Métodos:**

Uma pesquisa em banco de dados eletrônico foi realizada para RS e MA na Web of Science no dia 16 de julho de 2023, sem restrições de idioma e data de publicação. Dos 802 resultados encontrados, foram selecionados os 100 artigos ortodônticos mais citados. Eles foram analisados quanto a métricas de citação, características do periódico (revista, ano de publicação e fator de impacto [FI]), características dos autores e afiliação (quantidade, afiliação dos autores principal e correspondente, e país), domínio do estudo e palavras-chave.

**Resultados:**

Esses artigos foram publicados entre 1996 e 2021 em 20 periódicos com fator de impacto de 1,9 a 10,5, por 351 pesquisadores afiliados a 104 universidades. Suas citações variaram de 45 a 344, com 34 prestes a serem classificados como clássicos (≥ 100 citações). A maior quantidade de artigos foi publicada na *American Journal of Orthodontics and Dentofacial Orthopedics* (n=38), na *European Journal of Orthodontics (n=18),* e no *The Angle Orthodontist* (n=8). A quantidade de autores por artigo variou de 1 a 10, sendo 5 o mais frequente (n=58). A Europa teve a maior contribuição em relação ao número de autores correspondentes, instituições e citações. Ancoragem óssea e movimentação dentária ortodôntica/Biomecânica foram os domínios mais abordados (n=11 cada). A palavra-chave mais utilizada foi Ortodontia (n=19), seguida de Revisão Sistemática (n=16) e Meta-análise (n=9).

**Conclusão:**

No geral, as RS e MA mais citadas foram publicadas em revistas ortodônticas de alto impacto, eram de autoria múltipla e refletiam o trabalho colaborativo de diferentes universidades.

## INTRODUCTION

Bibliometric analysis is a scientific computer-assisted review methodology that identifies core research characteristics by covering all the publications related to a given topic or field. It typically measures research outputs like publication counts, citation counts, and measurements derived from these data.^1^ This information is a supporting tool for decision-making in setting research priorities, tracking the evolution of science and technology, funding allocation, and rewarding scientific excellence.^2^ One of the standard bibliometric methods is citation analysis, which quantifies the number and relationship of references an article receives over time.^3^ It also highlights the scientific progress and quality of research done throughout the years and the focus areas of active research. Despite being a time-dependent measure, it reflects the impact and progress of the research over the years, and is a widely used scientific quality indicator.^4^

With evidence-based clinical decision-making gaining momentum in all fields of Medicine and Dentistry, including Orthodontics, there is an increased focus on publications providing higher evidence.^5^ The widespread use of computer-based information systems and online access to publications have also enhanced the impact and utility of this evidence. According to the evidence-based Medicine pyramid, systematic review (SR) and meta-analysis (MA) provide the highest level of evidence, as they synthesize reliable information with varying levels of evidence from already available literature.^6^SRs are designed to answer specific questions by employing a predetermined, precise methodology to comprehensively search for, select, assess and analyze original research studies. SRs may or may not include formal MAs. MA is the statistical pooling of the results of studies that are part of a systematic review, and presents a significant advantage to SRs, by increasing the overall sample size by combining data from individual studies, thus increasing the statistical power and precision to assess the treatment effects.^7^

Citation analyses in Orthodontics have often focused on the time frame of publication or emerging domains within the field, such as Lingual Orthodontics,^8^ Temporary anchorage devices (TAD),^9^ etc. However, citation analysis of level-one evidence constituting SRs and MAs has not been attempted. Further, information on the bibliometric characteristics of impactful, high-quality publications will help understand the trends in synthetic research. Hence, the current study was designed to identify and analyze the bibliometric characteristics of the 100 most-cited SRs and MAs in Orthodontics. 

## MATERIAL AND METHODS

The Clarivate Analytics Web of Science (WoS) database has been widely used, being more accessible to historical literature. Therefore, a database search was performed in the WoS to track the top-cited SRs and MAs in Orthodontics. The search term in the topic field was entered as “(Systematic review OR Meta-analysis) AND Orthodont*” without any time and language restrictions. The systematic search was conducted on 16^th^ July 2023. The results were sorted based on the number of citations, and the first 200 articles were selected and exported to a Microsoft Excel spreadsheet (Microsoft Office 365, Microsoft Corporation, Redmond, Washington, USA), to evaluate their eligibility for inclusion in this study.

The preliminary screening of articles to assess their relevance to the study was performed independently by two authors (PV and US) based on the information from the title, abstract and the complete article, when required. Publications not directly related to orthodontics and those on craniofacial syndromes, cleft lip and palate were excluded. Any discordance related to the inclusion of a particular article was resolved by consensus discussion between all the authors. The hundred top-cited articles in the qualified list were included for data extraction and further analysis. 

The information retrieved included the total number of citations, the journal of publication, year of publication, number of authors, details of the primary and corresponding authors (affiliation, position, and country), funding organization, the journal’s impact factor (IF), Digital Object Identifier (DOI), study type and domain, and keywords.^8^ Manual data extraction and normalization were performed to unify terms and remove typographical errors. Normalization was carried out for the “Author,” “Organization,” and “Country of Origin” fields.^10^ In cases of multiple and different entries for the same author, their affiliations were verified and confirmed through an internet search. Only universities and higher research centers were noted for the study’s affiliation field. Information regarding departments, private practice, and smaller centers was not considered.^8^ ^,^ ^10^ The orthodontic study domains were classified as proposed by Aura-Tormos et al.^11^

Two investigators (MS and BN) independently collected and tabulated the data. After completion, they were compared for concurrence. A periodic team review was conducted to settle all discrepancies, and the consensus data thus obtained was treated as final. The data analysis and pictorial representation of data were developed using Microsoft Office 365 (Microsoft Corporation, Redmond, Washington, USA).

## RESULTS

**Figure 1:**
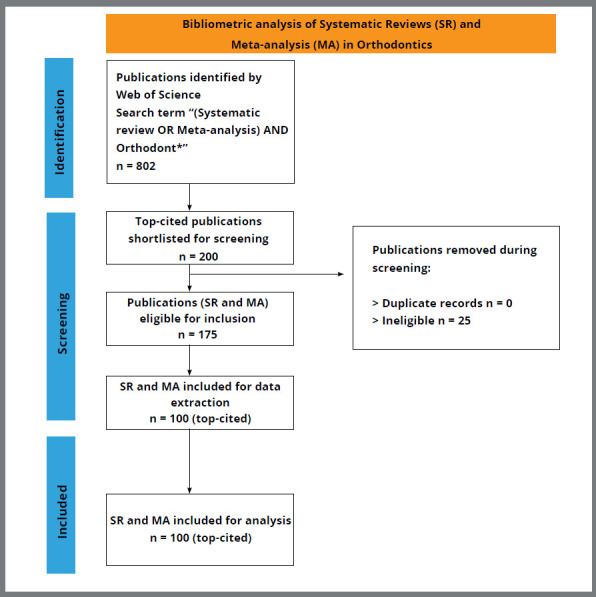
Flow chart depicting the inclusion of articles.

An initial keyword search yielded 802 articles. The top 200 were exported for scrutiny in descending order of the number of citations. Twenty-five articles not fulfilling the eligibility criteria regarding subject matter were eliminated during screening, leaving 175 for further consideration. From the final list, the 100 most-cited articles were included for analysis (Table 1). The systematic selection of articles is depicted in the flow chart (Fig 1). 

**Table 1:** Top 100 cited Systematic Review and Meta-analysis in Orthodontics.

Title	Authors	Reference	Year	Times cited, WoS core
Root resorption associated with orthodontic tooth movement: A systematic review	Weltman, B; Vig, KWL; Fields, HW; Shanker, S; Kaizar, EE	Am J Orthod Dentofacial Orthop. 2010;137(4):462-12A.	2010	344
Efficacy of clear aligners in controlling orthodontic tooth movement: A systematic review	Rossini, G; Parrini, S; Castroflorio, T; Deregibus, A; Debernardi, CL	Angle Orthod. 2015;85(5):881-889.	2015	277
Optimum force magnitude for orthodontic tooth movement: A systematic literature review	Ren, YJ; Maltha, JC; Kuijpers-Jagtman, AM	Angle Orthod. 2003;73(1):86-92.	2003	257
Orthodontic measurements on digital study models compared with plaster models: a systematic review	Fleming, PS; Marinho, V; Johal, A	Orthod Craniofac Res. 2011;14(1):1-16.	2011	219
Mandibular changes produced by functional appliances in Class II malocclusion: A systematic review	Cozza, P; Baccetti, T; Franchi, L; De Toffol, L; McNamara, JA	Am J Orthod Dentofacial Orthop. 2006;129(5):599.e1-e6.	2006	198
The effectiveness of protraction face mask therapy: A meta-analysis	Kim, JH; Viana, MAG; Graber, TM; Omerza, FF; BeGole, EA	Am J Orthod Dentofacial Orthop. 1999;115(6):675-685.	1999	191
Failure rates and associated risk factors of orthodontic miniscrew implants: A meta-analysis	Papageorgiou, SN; Zogakis, IP; Papadopoulos, MA	Am J Orthod Dentofacial Orthop. 2012;142(5):577-595.e7.	2012	190
Digital three-dimensional image fusion processes for planning and evaluating orthodontics and orthognathic surgery. A systematic review	Plooij, JM; Maal, TJJ; Haers, P; Borstlap, WA; Kuijpers-Jagtman, AM; Berge, SJ	Int J Oral Maxillofac Surg. 2011;40(4):341-352.	2011	188
The effects of orthodontic therapy on periodontal health - A systematic review of controlled evidence	Bollen, AM; Cunha-Cruz, J; Bakko, DW; Huang, GJ; Hujoel, PP	J Am Dent Assoc. 2008;139(4):413-422.	2008	169
The impact of malocclusion on the quality of life among children and adolescents: a systematic review of quantitative studies	Dimberg, L; Arnrup, K; Bondemark, L	Eur J Orthod. 2015;37(3):238-247.	2014	167
Skeletally anchored Forsus fatigue resistant device for correction of Class II malocclusions-A systematic review and meta-analysis	Arvind, TRP; Jain, RK	Orthod Craniofac Res. 2021;24(1):52-61.	2021	159
Orthodontic therapy and gingival recession: a systematic review	Joss-Vassalli, I; Grebenstein, C; Topouzelis, N; Sculean, A; Katsaros, C	Orthod Craniofac Res. 2010;13(3):127-141.	2010	150
Association of orthodontic force system and root resorption: A systematic review	Roscoe, MG; Meira, JBC; Cattaneo, PM	Am J Orthod Dentofacial Orthop. 2015;147(5):610-626.	2015	142
A systematic review of the relationship between overjet size and traumatic dental injuries	Nguyen, QV; Bezemer, PD; Habets, L; Prahl-Andersen, B	Eur J Orthod. 1999;21(5):503-515.	1999	142
Mini-implants in orthodontics: A systematic review of the literature	Reynders, R; Ronchi, L; Bipat, S	Am J Orthod Dentofacial Orthop. 2009;135(5):564.e1-565.	2009	139
Factors affecting the duration of orthodontic treatment: a systematic review	Mavreas, D; Athanasiou, AE	Eur J Orthod. 2008;30(4):386-395.	2008	139
Caries-inhibiting effect of preventive measures during orthodontic treatment with fixed appliances - A systematic review	Derks, A; Katsaros, C; Frencken, JE; van ‘t Hof, MA; Kuijpers-Jagtman, AM	Caries Res. 2004;38(5):413-420.	2004	139
Craniofacial structure and obstructive sleep apnea syndrome - A qualitative analysis and meta-analysis of the literature	Miles, PG; Vig, PS; Weyant, RJ; Forrest, TD; Rockette, HE	Am J Orthod Dentofacial Orthop. 1996;109(2):163-172.	1996	128
Craniofacial and upper airway morphology in pediatric sleep-disordered breathing: Systematic review and meta-analysis	Katyal, V; Pamula, Y; Martin, AJ; Daynes, CN; Kennedy, JD; Sampson, WJ	Am J Orthod Dentofacial Orthop. 2013;143(1):20-30.e3.	2013	127
How long does treatment with fixed orthodontic appliances last? A systematic review	Tsichlaki, A; Chin, SY; Pandis, N; Fleming, PS	Am J Orthod Dentofacial Orthop. 2016;149(3):308-318.	2016	125
Miniscrews in orthodontic treatment: Review and analysis of published clinical trials	Crismani, AG; Bertl, MH; Celar, AG; Bantleon, HP; Burstone, CJ	Am J Orthod Dentofacial Orthop. 2010;137(1):108-113.	2010	120
Medication effects on the rate of orthodontic tooth movement: A systematic literature review	Bartzela, T; Tuerp, JC; Motschall, E; Maltha, JC	Am J Orthod Dentofacial Orthop. 2009;135(1):16-26.	2009	119
Retention procedures for stabilising tooth position after treatment with orthodontic braces	Littlewood, SJ; Millett, DT; Doubleday, B; Bearn, DR; Worthington, HV	Cochrane Database Syst Rev. 2016;2016(1):CD002283.	2016	117
Stability of treatment for anterior open-bite malocclusion: A meta-analysis	Greenlee, GM; Huang, GJ; Chen, SSH; Chen, JD; Koepsell, T; Hujoel, P	Am J Orthod Dentofacial Orthop. 2011;139(2):154-169.	2011	114
Accuracy, reliability, and efficiency of intraoral scanners for full-arch impressions: a systematic review of the clinical evidence	Goracci, C; Franchi, L; Vichi, A; Ferrari, M	Eur J Orthod. 2016;38(4):422-428.	2015	112
The orthodontic-periodontic interrelationship in integrated treatment challenges: a systematic review	Gkantidis, N; Christou, P; Topouzelis, N	J Oral Rehabil. 2010;37(5):377-390.	2010	112
Critical factors for the success of orthodontic mini-implants: A systematic review	Chen, Y; Kyung, HM; Zhao, WT; Yu, WJ	Am J Orthod Dentofacial Orthop. 2009;135(3):284-291.	2009	112
Diagnostic accuracy and measurement sensitivity of digital models for orthodontic purposes: A systematic review	Rossini, G; Parrini, S; Castroflorio, T; Deregibus, A; Debernardi, CL	Am J Orthod Dentofacial Orthop. 2016;149(2):161-170.	2016	109
Interventions for accelerating orthodontic tooth movement A systematic review	Long, H; Pyakurel, U; Wang, Y; Liao, LN; Zhou, Y; Lai, WL	Angle Orthod. 2013;83(1):164-171.	2013	107
TMD in relation to malocclusion and orthodontic treatment - A systematic review	Mohlin, B; Axelsson, S; Paulin, G; Pietila, T; Bondemark, L; Brattstrom, V; Hansen, K; Holm, AK	Angle Orthod. 2007;77(3):542-548.	2007	107
Validity and reliability of intraoral scanners compared to conventional gypsum models measurements: a systematic review	Aragon, MLC; Pontes, LF; Bichara, LM; Flores-Mir, C; Normando, D	Eur J Orthod. 2016;38(4):429-434.	2016	106
Cephalometric landmarks identification and reproducibility: A meta analysis	Trpkova, B; Major, P; Prasad, N; Nebbe, B	Am J Orthod Dentofacial Orthop. 1997;112(2):165-170.	1997	104
Assessment of lateral cephalometric diagnosis of adenoid hypertrophy and posterior upper airway obstruction: A systematic review	Major, MP; Flores-Mir, C; Major, PW	Am J Orthod Dentofacial Orthop. 2006;130(6):700-708.	2006	103
Clinical effectiveness of Invisalign (R) orthodontic treatment: a systematic review	Papadimitriou, A; Mousoulea, S; Gkantidis, N; Kloukos, D	Prog Orthod. 2018;19(1):37.	2018	101
Cytokines in crevicular fluid and orthodontic tooth movement	Ren, YJ; Vissink, A	Eur J Oral Sci. 2008;116(2):89-97.	2008	99
Self-Ligating Brackets in Orthodontics A Systematic Review	Fleming, PS; Johal, A	Angle Orthod. 2010;80(3):575-584.	2010	98
Systematic review of self-ligating brackets	Chen, SSH; Greenlee, GM; Kim, JE; Smith, CL; Huang, GJ	Am J Orthod Dentofacial Orthop. 2010;137(6):726.e1-727.	2010	96
Treatment effects of fixed functional appliances in patients with Class II malocclusion: a systematic review and meta-analysis	Zymperdikas, VF; Koretsi, V; Papageorgiou, SN; Papadopoulos, MA	Eur J Orthod. 2016;38(2):113-126.	2015	95
Treatment effects of removable functional appliances in patients with Class II malocclusion: a systematic review and meta-analysis	Koretsi, V; Zymperdikas, VF; Papageorgiou, SN; Papadopoulos, MA	Eur J Orthod. 2015;37(4):418-434.	2014	93
Correction of Class II malocclusion with Class II elastics: A systematic review	Janson, G; Sathler, R; Fernandes, TMF; Branco, NCC; de Freitas, MR	Am J Orthod Dentofacial Orthop. 2013;143(3):383-392.	2013	89
The treatment effects of invisalign orthodontic aligners - A systematic review	Lagravere, MO; Flores-Mir, C	J Am Dent Assoc. 2005;136(12):1724-1729.	2005	89
Systematic review of the experimental use of temporary skeletal anchorage devices in orthodontics	Cornelis, MA; Scheffler, NR; De Clerck, HJ; Tulloch, JFC; Nyssen-Behets, C	Am J Orthod Dentofacial Orthop. 2007;131(4 Suppl):S52-S58.	2007	87
A meta-analysis of mandibular intercanine width in treatment and postretention	Burke, SP; Silveira, AM; Goldsmith, LJ; Yancey, JM; Van Stewart, A; Scarfe, WC	Angle Orthod. 1998;68(1):53-60.	1998	87
Periodontal health during clear aligners treatment: a systematic review	Rossini, G; Parrini, S; Castroflorio, T; Deregibus, A; Debernardi, CL	Eur J Orthod. 2015;37(5):539-543.	2014	82
Efficiency, effectiveness and treatment stability of clear aligners: A systematic review and meta-analysis	Zheng, M; Liu, R; Ni, Z; Yu, Z	Orthod Craniofac Res. 2017;20(3):127-133.	2017	81
Rapid Maxillary Expansion for Pediatric Obstructive Sleep Apnea: A Systematic Review and Meta-Analysis	Camacho, M; Chang, ET; Song, SJA; Abdullatif, J; Zaghi, S; Pirelli, P; Certal, V; Guilleminault, C	Laryngoscope. 2017;127(7):1712-1719.	2016	81
Does rapid maxillary expansion have long-term effects on airway dimensions and breathing?	Baratieri, C; Alves, M; de Souza, MMG; Araujo, MTD; Maia, LC	Am J Orthod Dentofacial Orthop. 2011;140(2):146-156.	2011	80
Does orthodontic treatment before the age of 18 years improve oral health-related quality of life? A systematic review and meta-analysis	Javidi, H; Vettore, M; Benson, PE	Am J Orthod Dentofacial Orthop. 2017;151(4):644-655.	2017	79
Effectiveness of clear aligner therapy for orthodontic treatment: A systematic review	Robertson, L; Kaur, H; Fagundes, NCF; Romanyk, D; Major, P; Mir, CF	Orthod Craniofac Res. 2020;23(2):133-142.	2020	77
Orthodontics and temporomandibular disorder: A meta-analysis	Kim, MR; Graber, TM; Viana, MA	Am J Orthod Dentofacial Orthop.2002 May;121(5):438-46.	2002	77
Factors associated with patient and parent satisfaction after orthodontic treatment: A systematic review	Pacheco-Pereira, C; Pereira, JR; Dick, BD; Perez, A; Flores-Mir, C	Am J Orthod Dentofacial Orthop. 2015;148(4):652-9	2015	76
Evidence supporting the use of cone-beam computed tomography in orthodontics	van Vlijmen, OJC; Kuijpers, MAR; Berge, SJ; Schols, JGJH; Maal, TJJ; Breuning, H; Kuijpers-Jagtman, AM	J Am Dent Assoc.2012 Mar;143(3):241-52	2012	76
A systematic review of the efficacy of oral appliance design in the management of obstructive sleep apnoea	Ahrens, A; McGrath, C; Hagg, U	Eur J Orthod,2011 Jun;33(3):318-24.	2011	74
Efficacy of orthopedic treatment with protraction facemask on skeletal Class III malocclusion: a systematic review and meta-analysis	Cordasco, G; Matarese, G; Rustico, L; Fastuca, S; Caprioglio, A; Lindauer, SJ; Nucera, R	Orthod Craniofac Res. 2014 Aug;17(3):133-43	2014	73
Influence of orthodontic treatment, midline position, buccal corridor and smile arc on smile attractiveness A systematic review	Janson, G; Branco, NC; Fernandes, TMF; Sathler, R; Garib, D; Lauris, JRP	Angle Orthod. 2011 Jan;81(1):153-61	2011	71
A comparison of treatment effectiveness between clear aligner and fixed appliance therapies	Ke, YY; Zhu, YF; Zhu, M	BMC Oral Health. 2019 Jan 23;19(1):24	2019	69
Effectiveness of non-conventional methods for accelerated orthodontic tooth movement: A systematic review and meta-analysis	Gkantidis, N; Mistakidis, I; Kouskoura, T; Pandis, N	J Dent. 2014 Oct;42(10):1300-19	2014	69
Compliance with removable orthodontic appliances and adjuncts: A systematic review and meta-analysis	Al-Moghrabi, D; Salazar, FC; Pandis, N; Fleming, PS	Am J Orthod Dentofacial Orthop . 2017 Jul;152(1):17-32.	2017	68
Surgically facilitated orthodontic treatment: A systematic review	Hoogeveen, EJ; Jansma, J; Ren, Y	Am J Orthod Dentofacial Orthop. 2014 Apr;145(4 Suppl):S51-64.	2014	68
Interventions for orthodontically induced white spot lesions: a systematic review and meta-analysis	Hochli, D; Hersberger-Zurfluh, M; Papageorgiou, SN; Eliades, T	Eur J Orthod. 2017 Apr 1;39(2):122-133	2017	67
Effectiveness of orthodontic miniscrew implants in anchorage reinforcement during en-masse retraction: A systematic review and meta-analysis	Antoszewska-Smith, J; Sarul, M; Lyczek, J; Konopka, T; Kawala, B	Am J Orthod Dentofacial Orthop . 2017 Mar;151(3):440-455.	2017	65
Effectiveness of minimally invasive surgical procedures in the acceleration of tooth movement: a systematic review and meta-analysis	Alfawal, AMH; Hajeer, MY; Ajaj, MA; Hamadah, O; Brad, B	Prog Orthod. 2016 Dec;17(1):33	2016	64
Orthodontics for treating temporomandibular joint (TMJ) disorders	Luther, F; Layton, S; McDonald, F	Cochrane Database Syst Rev. 2010 Jul 7;(7):CD006541	2010	64
Early orthodontic treatment for Class III malocclusion: A systematic review and meta-analysis	Woon, SC; Thiruvenkatachari, B	Am J Orthod Dentofacial Orthop . 2017 Jan;151(1):28-52	2017	63
Orthodontics treatments for managing obstructive sleep apnea syndrome in children: A systematic review and meta-analysis	Huynh, NT; Desplats, E; Almeida, FR	Sleep Med Rev . 2016 Feb;25:84-94	2016	63
Effects of rapid maxillary expansion on the midpalatal suture: a systematic review	Liu, SY; Xu, TM; Zou, W	Eur J Orthod . 2015 Dec;37(6):651-5	2015	63
Do orthodontic research outcomes reflect patient values? A systematic review of randomized controlled trials involving children	Tsichlaki, A; O’Brien, K	Am J Orthod Dentofacial Orthop . 2014 Sep;146(3):279-85.	2014	63
The impact of orthodontic treatment on the quality of life a systematic review	Zhou, Y; Wang, Y; Wang, XY; Voliere, G; Hu, RD	BMC Oral Health. 2014 Jun 10;14:66	2014	63
A systematic review of the accuracy and efficiency of dental movements with Invisalign (R)	Galan-Lopez, L; Barcia-Gonzalez, J; Plasencia, E	Korean J Orthod. 2019 May; 49(3): 140-149	2019	62
In-vitro orthodontic bond strength testing: A systematic review and meta-analysis	Finnema, KJ; Ozcan, M; Post, WJ; Ren, YJ; Dijkstra, PU	Am J Orthod Dentofacial Orthop . 2010 May;137(5):615-622.e3	2010	62
Available Technologies, Applications and Benefits of Teleorthodontics. A Literature Review and Possible Applications during the COVID-19 Pandemic	Maspero, C; Abate, A; Cavagnetto, D; El Morsi, M; Fama, A; Farronato, M	J Clin Med . 2020 Jun 17;9(6):1891.	2020	60
Insertion torque and success of orthodontic mini-implants: A systematic review	Reynders, RAM; Ronchi, L; Ladu, L; van Etten-Jamaludin, F; Bipat, S	Am J Orthod Dentofacial Orthop . 2012 Nov;142(5):596-614.e5	2012	60
Effectiveness of orthodontic treatment with functional appliances on mandibular growth in the short term	Marsico, E; Gatto, E; Burrascano, M; Matarese, G; Cordasco, G	Am J Orthod Dentofacial Orthop . 2011 Jan;139(1):24-36	2011	59
Corticotomies and Orthodontic Tooth Movement: A Systematic Review	Patterson, BM; Dalci, O; Darendeliler, MA; Papadopoulou, AK	J Oral Maxillofac Surg . 2016 Mar;74(3):453-73.	2016	57
Bone age assessment with various machine learning techniques: A systematic literature review and meta-analysis	Dallora, AL; Anderberg, P; Kvist, O; Mendes, E; Ruiz, SD; Berglund, JS	Plos one :2019 Jul 25;14(7):e0220242.	2019	55
Comparison of vacuum-formed and Hawley retainers: A systematic review	Mai, WJ; He, JA; Meng, HY; Jiang, YP; Huang, CX; Li, M; Yuan, K; Kang, N	Am J Orthod Dentofacial Ortho 2014 Jun;145(6):720-7.	2014	55
Treatment outcome with orthodontic aligners and fixed appliances: a systematic review with meta-analyses	Papageorgiou, SN; Koletsi, D; Iliadi, A; Peltomaki, T; Eliades, T	Eur J Orthod : 2020 Jun 23;42(3):331-343	2019	54
Effect of remineralizing agents on white spot lesions after orthodontic treatment: A systematic review	Chen, H; Liu, XG; Dai, J; Jiang, ZW; Guo, T; Ding, Y	Am J Orthod Dentofacial Orthop : 2013 Mar;143(3):376-382.e3.	2013	54
Effects of malocclusions and orthodontics on periodontal health: Evidence from a systematic review	Bollen, AM	J Am Dent Assoc 2008 Apr;139(4):413-22.	2008	54
Laypeople’s perceptions of frontal smile esthetics: A systematic review	Parrini, S; Rossini, G; Castroflorio, T; Fortini, A; Deregibus, A; Debernardie, C	Am J Orthod Dentofacial Orthop : 2016 Nov;150(5):740-750	2016	53
Determinants for success rates of temporary anchorage devices in orthodontics: a meta-analysis (n > 50)	Dalessandri, D; Salgarello, S; Dalessandri, M; Lazzaroni, E; Piancino, M; Paganelli, C; Maiorana, C; Santoro, F	Eur J Orthod : 2014 Jun;36(3):303-13	2013	52
The effects of fixed and removable orthodontic retainers: a systematic review	Al-Moghrabi, D; Pandis, N; Fleming, PS	Prog Orthod : 2016 Dec;17(1):24	2016	51
Intra-arch dimensional measurement validity of laser-scanned digital dental models compared with the original plaster models: a systematic review	Canto, GD; Pacheco-Pereira, C; Lagravere, MO; Flores-Mir, C; Major, PW	Orthod Craniofac Res 2015 May;18(2):65-76	2014	51
Three-dimensional cephalometric analysis in orthodontics: a systematic review	Pittayapat, P; Limchaichana-Bolstad, N; Willems, G; Jacobs, R	Orthod Craniofac Res 2014 May;17(2):69-91	2014	51
Lingual vs. labial fixed orthodontic appliances: systematic review and meta-analysis of treatment effects	Papageorgiou, SN; Golz, L; Jager, A; Eliades, T; Bourauel, C	Eur J Oral Sci 2016 Apr;124(2):105-18.	2016	50
Efficacy of professional hygiene and prophylaxis on preventing plaque increase in orthodontic patients with multibracket appliances: a systematic review	Migliorati, M; Isaia, L; Cassaro, A; Rivetti, A; Silvestrini-Biavati, F; Gastaldo, L; Piccardo, I; Dalessandri, D; Silvestrini-Biavati, A	Eur J Orthod 2015 Jun;37(3):297-307	2014	50
Management of post-orthodontic white spot lesions: an updated systematic review	Sonesson, M; Bergstrand, F; Gizani, S; Twetman, S	Eur J Orthod. 2017 Apr 1;39(2):116-121.	2017	49
Intraoral distalizer effects with conventional and skeletal anchorage: A meta-analysis	Grec, RHD; Janson, G; Branco, NC; Moura-Grec, PG; Patel, MP; Henriques, JFC	Am J Orthod Dentofacial Orthop 2013 May;143(5):602-15.	2013	49
Bisphenol-A and residual monomer leaching from orthodontic adhesive resins and polycarbonate brackets: A systematic review	Kloukos, D; Pandis, N; Eliades, T	Am J Orthod Dentofacial Orthop 2013 Apr;143(4 Suppl):S104-12.e1-2.	2013	49
CBCT in orthodontics: a systematic review on justification of CBCT in a paediatric population prior to orthodontic treatment	De Grauwe, A; Ayaz, I; Shujaat, S; Dimitrov, S; Gbadegbegnon, L; Vande Vannet, B; Jacobs, R	Eur J Orthod 2019 Aug 8;41(4):381-389	2018	48
Miniscrews failure rate in orthodontics: systematic review and meta-analysis	Alharbi, F; Almuzian, M; Bearn, D	Eur J Orthod 2018 Sep 28;40(5):519-530.	2018	48
Systematic review for orthodontic and orthopedic treatments for anterior open bite in the mixed dentition	Pisani, L; Bonaccorso, L; Fastuca, R; Spena, R; Lombardo, L; Caprioglio, A	Prog Orthod 2016 Dec;17(1):28.	2016	48
Anterior cranial-base time-related changes: A systematic review	Afrand, M; Ling, CP; Khosrotehrani, S; Flores-Mir, C; Lagravere-Vich, MO	Am J Orthod Dentofacial Orthop 2014 Jul;146(1):21-32.e6.	2014	48
Clinical effects of pre-adjusted edgewise orthodontic brackets: a systematic review and meta-analysis	Papageorgiou, SN; Konstantinidis, I; Papadopoulou, K; Jager, A; Bourauel, C	Eur J Orthod 2014 Jun;36(3):350-63	2013	48
Orthodontic treatment for prominent upper front teeth (Class II malocclusion) in children	Thiruvenkatachari, B; Harrison, JE; Worthington, HV; O’Brien, KD	Cochrane Database Syst Rev 2013 Nov 13;(11):CD003452	2014	48
Role of anatomical sites and correlated risk factors on the survival of orthodontic miniscrew implants: a systematic review and meta-analysis	Mohammed, H; Wafaie, K; Rizk, MZ; Almuzian, M; Sosly, R; Bearn, DR	Prog Orthod 2018 Sep 24;19(1):36.	2018	47
Surgical adjunctive procedures for accelerating orthodontic treatment	Fleming, PS; Fedorowicz, Z; Johal, A; El-Angbawi, A; Pandis, N	Cochrane Database Syst Rev 2015 Jun 30;2015(6):CD010572.	2016	46
The effect of topical fluorides on decalcification in patients with fixed orthodontic appliances: A systematic review	Chadwick, BL; Roy, J; Knox, J; Treasure, ET	Am J Orthod Dentofacial Orthop. 2005;128(5):601-6.	2005	46
Orthodontic treatment for posterior crossbites	Agostino, P; Ugolini, A; Signori, A; Silvestrini-Biavati, A; Harrison, JE; Riley, P	Cochrane Database Syst Rev. 2014 Aug 8;(8):CD000979.	2014	45
Comparison of adverse effects between lingual and labial orthodontic treatment A systematic review	Long, H; Zhou, Y; Pyakurel, U; Liao, LN; Jian, F; Xue, JJ; Ye, NS; Yang, X; Wang, Y; Lai, WL	Angle Orthod 2013 Nov;83(6):1066-73.	2013	45

### CITATION METRICS

**Figure 2:**
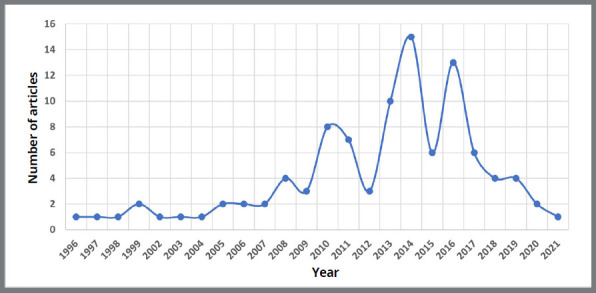
Top 100 cited systematic review and meta-analysis in orthodontics over the years.

The citation counts of the top 100 cited articles ranged from 45 to 344. These articles were published from 1996 to 2021 (Fig 2), with spikes in 2014 (n=15) and 2016 (n=13). Of these, 59 were SR, 6 were MA, and 35 were SR with MA. Thirty-four articles were cited more than 100 times and considered classic articles. The article *“Root resorption associated with orthodontic tooth movement: A systematic review”,* from American Journal of Orthodontics and Dentofacial Orthopedics (AJODO), was the most cited publication. 

### JOURNAL CHARACTERISTICS

The top-cited articles were published in 20 different scientific journals (Table 2). Among these, were six journals specialized in Orthodontics, which collectively published more than three-fourths of the publications (n=78) of the entire lot. The maximum number of articles (n=38) were published in AJODO (IF=3), with a total citation of 3,811. Of these, 23 were SR, 4 were MA, and the rest were SR with MA. There were notable publications in the AJODO in 2013 (n=5). Meanwhile, the Angle Orthodontist had the highest citation/article ratio (131.13). The least cited article among the top 100 was published in the Journal of Dental Education (IF=2.3). The IF of the journals ranged from 1.9 to 10.5 (3.4 ± 1.4, median = 3).

**Table 2:** The 20 journals in which the top 100 cited articles published.

Nº	Journal title	ISSN	Journal impact factor	Number of articles	Systematic reviews(SR)	Meta-analysis (MA)	SR with MA	Total citations	Citation/Article
1	American Journal of Orthodontics and Dentofacial Orthopedics	0889-5406	3	38	23	4	11	3811	100.29
2	European Journal of Orthodontics	0141-5387	2.6	18	11	1	6	1489	82.72
3	Angle Orthodontist	0003-3219	3.4	8	4	1	3	1049	131.13
4	Orthodontics & Craniofacial Research	1601-6335	3.1	8	5	-	3	861	107.63
5	Cochrane Database of Systematic Reviews	1469-493X	8.4	5	2	-	3	320	64.00
6	Progress in Orthodontics	2196-1042	4.8	5	2	-	3	311	62.20
7	Journal of The American Dental Association	0002-8177	3.9	3	3	-	-	334	111.33
8	European Journal Of Oral Sciences	0909-8836	1.9	2	1	-	1	149	74.50
9	BMC Oral Health	1472-6831	2.9	2	1	-	1	132	66
10	International Journal of Oral and Maxillofacial Surgery	0901-5027	2.4	1	1	-	-	188	188
11	Caries Research	0008-6568	3.9	1	1	-	-	139	139
12	Journal of Oral Rehabilitation	0305-182X	2.9	1	1	-	-	112	112
13	Laryngoscope	0023-852X	2.6	1	-	-	1	81	81
14	Journal of Dentistry	0300-5712	4.4	1	-	-	1	69	69
15	Sleep Medicine Reviews	1087-0792	10.5	1	-	-	1	63	63
16	Korean Journal of Orthodontics	2234-7518	1.9	1	1	-	-	62	62
17	Journal of Clinical Medicine		3.9	1	1	-	-	60	60
18	Journal of Oral and Maxillofacial Surgery	0278-2391	1.9	1	1	-	-	57	57
19	PLOS One	1932-6203	3.7	1	-	-	1	55	55
20	Journal of Dental Education	0022-0337	2.3	1	1	-	-	54	54

### AUTHOR CHARACTERISTICS

The top 100 cited articles were co-authored by 351 authors from different countries and universities. Publications with more than five authors were the most common (n=58) (Supplementary table). Sixty-two authors have contributed to two or more articles (Table 3). The number of authors for individual papers ranged from 1 to 10. Kuijpers-Jagtman AM, from Radboud University Nijmegen (The Netherlands), had the highest number of individual citations (n=660), from four research papers.

**Table 3:** The top-cited authors with two or more publications.

Nº	Author name	Affiliation	Country	Number of articles	Number of articles as first author	Number of articles as corresponding author	Total number of citations
1	Kuijpers-Jagtman, AM	Radboud University Nijmegen	The Netherlands	4	-	-	660
2	Fleming, PS	Queen Mary University of London	United Kingdom	6	3	4	607
3	Papageorgiou, SN	University of Bonn	Germany	7	4	2	597
4	Flores-Mir, C	University of Alberta	Canada	7	2		550
5	Castroflorio, T	University of Turin	Italy	4	-	-	521
6	Debernardi, CL	University of Turin	Italy	4	-	-	521
7	Deregibus, A	University of Turin	Italy	4	-	-	521
8	Parrini, S	University of Turin	Italy	4	-	-	521
9	Rossini, G	University of Turin	Italy	4	3	3	521
10	Ren, Y	University of Groningen	The Netherlands	4	2	2	486
11	Pandis, N	University of Bern	Switzerland	6	-	-	408
12	Huang, GJ	University of Washington	United States	3	-	-	379
13	Maltha, JC	Radboud University Nijmegen	The Netherlands	2	-	-	376
14	Johal, A	Queen Mary University of London	United Kingdom	3	-	-	363
15	Major, PW	University of Alberta	Canada	4	-	-	335
16	Franchi, L	University of Florence	Italy	2	-	-	310
17	Katsaros, C	Radboud University Nijmegen	The Netherlands	2	-	-	289
18	Papadopoulos, MA	Aristotle University of Thessaloniki	Greece	3	-	3	285
19	Hujoel, P	University of Washington	United States	2	-	-	283
20	Bondemark, L	Malmo University	Sweden	2	-	-	274
21	Graber, TM	University of Illinois	United States	2	-	-	268
22	Viana, MA	University of Illinois	United States	2	-	-	268
23	Berge, SJ	Radboud University Nijmegen	The Netherlands	2	-	-	264
24	Maal, TJJ	Radboud University Nijmegen	The Netherlands	2	-	-	264
25	Topouzelis, N	Aristotle University of Thessaloniki	Greece	2	-	-	262
26	Bollen, AM	University of Washington	United States	2	2	2	223
27	Eliades, T	University of Zurich	Switzerland	4	-	3	220
28	Gkantidis, N	University of Bern	Switzerland	3	2	-	213
29	Bearn, D	University of Dundee	United Kingdom	3	-	-	212
30	Chen, SSH	University of Washington	United States	2	-	-	210
31	Greenlee, GM	University of Washington	United States	2	-	-	210
32	Branco, NC	University of Sao Paulo	Brazil	3	-	-	209
33	Janson, G	University of Sao Paulo	Brazil	3	2	2	209
34	Bipat, S	University of Amsterdam	The Netherlands	2	-	-	199
35	Reynders, R	Private practice	Italy	2	-	2	199
36	Ronchi, L	University of Amsterdam	The Netherlands	2	-	-	199
37	Koretsi, V	University of Regensburg	Greece	2	-	-	188
38	Lagravere, MO	University of Alberta	Canada	3	-	-	188
39	Tsichlaki, A	Queen Mary University of London	United Kingdom	2	-	-	188
40	Zymperdikas, VF	Dental Unit,71st Airmobile Brigade	Greece	2	-	-	188
41	Worthington, HV	University of Manchester	United Kingdom	2	-	-	165
42	Fernandes, TMF	University of Sao Paulo	Brazil	2	-	-	160
43	Sathler, R	University of Sao Paulo	Brazil	2	-	-	160
44	Wang, Y	Sichuan University	China	2	-	-	152
45	Zhou, Y	Sichuan University	China	2	-	-	152
46	Lai, WL	Sichuan University	China	2	-	2	152
47	Liao, LN	Sichuan University	China	2	-	-	152
48	Long, H	Sichuan University	China	2	2	-	152
49	Pyakurel, U	Sichuan University	China	2	-	-	152
50	Kloukos, D	University of Bern	Switzerland	2	-	-	150
51	Cordasco, G	University of Messina	Italy	2	-	-	132
52	Pacheco-Pereira, C	University of Alberta	Canada	2	-	-	127
53	Caprioglio, A	University of Insubria	Italy	2	-	-	121
54	Al-Moghrabi, D	Queen Mary University of London	United Kingdom	2	2	2	119
55	O'Brien, K	University of Manchester	United Kingdom	2	-	-	111
56	Thiruvenkatachari, B	University of Manchester	United Kingdom	2	-	2	111
57	Dalessandri, D	University of Brescia	Italy	2	-	-	102
58	Jacobs, R	University of Leuven	Belgium	2	-	-	99
59	Bourauel, C	University of Bonn	Germany	2	-	-	98
60	Jager, A	University of Bonn	Germany	2	-	-	98
61	Almuzian, M	University of Leuven	Belgium	2	-	-	95
62	Silvestrini-Biavati, A	University of Genoa	Italy	2	-	-	95

Individually, Papageorgiou SN and Flores-Mir C, affiliated with the University of Bonn (Germany) and the University of Alberta (Canada), respectively, co-authored a maximum of seven papers. In addition, Papageorgiou SN contributed to a maximum number of four articles as a first author. Similarly, Fleming PS, affiliated with the Queen Mary University of London (United Kingdom), had a maximum of four articles as the corresponding author.

### AUTHOR AFFILIATION AND COUNTRY

Among 104 universities associated with these top-cited articles, 33 were affiliated with two or more articles (Table 4). The highest number of individual citations (n=929) was by Radboud University Nijmegen (The Netherlands). The Aristotle University of Thessaloniki (Greece), the University of Bern (Switzerland), and the University of Alberta (Canada) contributed with eight articles each. 

**Table 4:** The top 33 universities with two or more articles.

Nº	Affiliation/University	Country	Number of articles	Number of citations
1	Radboud University Nijmegen	The Netherlands	6	929
2	Aristotle University of Thessaloniki	Greece	8	905
3	University of Bern	Switzerland	8	659
4	University of Alberta	Canada	8	654
5	University of Turin	Italy	6	623
6	Queen Mary University of London	United Kingdom	6	607
7	University of Bonn	Germany	6	543
8	University of Washington	United States	4	433
9	University of São Paulo	Brazil	4	351
10	University of Amsterdam	The Netherlands	3	341
11	Malmo University	Sweden	3	323
12	University of Florence	Italy	2	310
13	University of Manchester	United Kingdom	4	291
14	University of Zurich	Switzerland	5	282
15	University of Rome Tor Vergata	Italy	2	279
16	Catholic University of Korea	South Korea	2	268
17	University of Illinois	United States	2	268
18	University of Dundee	United Kingdom	4	258
19	University of Sydney	Australia	3	232
20	University of Groningen	The Netherlands	3	229
21	National & Kapodistrian University of Athens	Greece	3	215
22	University of Regensburg	Germany	2	188
23	Sichuan University	China	2	152
24	Wenzhou Medical University	China	2	144
25	University of Messina	Italy	2	132
26	University of Insubria	Italy	2	121
27	University of Geneva	Switzerland	2	116
28	University of Milan	Italy	2	112
29	Karolinska Institute	Sweden	2	103
30	University of Brescia	Italy	2	102
31	University of Leuven	Belgium	2	99
32	University of Genoa	Italy	2	95
33	Liverpool University	United Kingdom	2	93

**Figure 3:**
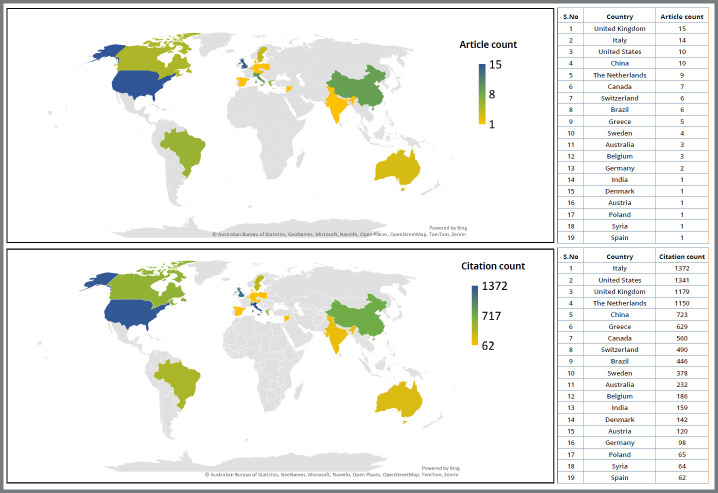
The country-wise distribution of corresponding authors and citations among the top 100 cited articles.

Based on the article’s corresponding author’s origin, these publications came from 19 countries. Figure 3 (World map, Microsoft Office 365, Microsoft Corporation, Redmond, Washington, USA) depicts the distribution of corresponding authors and citation analysis. The maximum number of citations (n=1372) contributed by corresponding authors belonged to Italy, from 14 research articles. However, the United Kingdom published the maximum number of articles (n=15) with a citation of 1179. In addition, 62 corresponding authors with two or more of the top cited articles were from 12 countries from the European continent.

### STUDY DOMAIN

There was a wide distribution of articles based on research domains in Orthodontics. A total of 21 domains were discussed among the top 100 cited systematic reviews and meta-analyses (Table 5). The most focused domains were orthodontic tooth movement (OTM) / biomechanics (11 articles, 1150 citations) and bone anchorage (11 articles, 969 citations). This was followed by digital Orthodontics, Class II management with functional appliances, and Aligners. 

**Table 5:** Distribution of articles, based on research domains.

Nº	Domain	Number of articles	Total citation
1	Orthodontic tooth movement/Biomechanics	11	1150
2	Bone anchorage	11	969
3	Digital orthodontics	6	785
4	Class II fixed or removable functional appliances	7	741
5	Aligners	7	729
6	Stability and relapse/retention/fixed and removable retainers	7	573
7	Psychological and psychosocial aspects in patients	7	572
8	Orthodontic-periodontal consideration	5	567
9	Upper airways and sleep apnea	5	495
10	Root resorption	2	486
11	Bracket design, friction, self-ligating	5	337
12	Imaging/Cephalometrics/CBCT	5	327
13	Class III Orthopedics	3	327
14	Transverse discrepancy/Maxillary expansion	4	269
15	TMJ and craniomandibular dysfunction	3	248
16	Demineralization, White Spot lesion	4	216
17	Preventive measure	2	189
18	Others: Adhesive resins	3	164
19	Dental trauma	1	142
20	Bonding and bracket removal	1	62
21	Vertical discrepancy	1	48

**Table 6:** Most commonly used Keywords in the top 100 cited articles.

Nº	Keywords	Number of times
1	Orthodontics	19
2	Systematic review	16
3	Meta-analysis	9
4	Clear aligner	6
5	Treatment outcome	6
6	Periodontal	5
7	Child	4
8	Human study	4
9	Invisalign	4
10	Malocclusion	4
11	Orthodontic retainer	4
12	Corticotomy	3
13	Fixed appliance	3
14	Orthodontic appliances	3
15	Orthodontics, Corrective [methods]	3
16	Randomized Controlled Trials as topic	3
17	Review	3

The top five cited articles were mostly related to root resorption (n=344), clear aligners (n=277), OTM (n=257), digital study models (n=219), and functional appliances (n=198). With the recent advances in digital Orthodontics, aligners, and fixed functional appliances, the volume of research with citations has increased significantly. Domains like dental trauma, vertical discrepancy, and bonding and bracket removal presented least number of articles with citations. Citation analysis of journals regarding the domain bone anchorage/mini-implant (n=9) showed that these articles were most commonly published in AJODO from 2007 to 2017 (Supplementary Table).

### KEYWORDS

There were 212 unique keywords provided by 40 research papers in the lot. The most frequent were Orthodontics (n=19), Systematic review (n=16) and Meta-analysis (n=9). Details of keywords used thrice or more are presented in Table 6.

## DISCUSSION

Citation metric is a popular quantitative measure of the impact of a research article in a particular domain. Bibliometric studies analyzing various aspects of this metric and its associated factors have been a common practice in many specialties of Dentistry. In Orthodontics, scientific mapping has been conducted in many areas, like Lingual Orthodontics,^8^ TAD,^9^ Orthognathic Surgery,^12^ and Artificial Intelligence.^13^ However, bibliometric studies on level one evidence articles are unavailable; to the best of our knowledge, this is the first in this regard.

Overall, the articles covered 21 subject domains. Orthodontic tooth movement/Biomechanics topped the list, with 1150 citations from 11 articles. This finding is not surprising, as this is a fundamental domain for the practice of Orthodontics. Three of the top five domains were related to recent evolutions in Orthodontics: Bone anchorage, Digital orthodontics, and Aligners. As recent advances in any field exhibit a higher knowledge gap, evoke more interest, and instigate more research and publications, it is natural to note increasing citations of seminal publications in the domain.^14^ Among individual articles, the systematic review on root resorption by Weltman et al.,^15^ published by AJODO in 2010, topped the number of citations (n=344). This article reported high-quality evidence of risk factors associated with root resorption associated with fixed orthodontics. It should also be noted that nearly one-fourth of the articles were published in journals not exclusive to the specialty of Orthodontics, highlighting the need to go beyond specialty journals while searching for relevant content. 

The scientific literature on Orthodontics is vast, and articles reaching over 100 citations are considered highly impactful and classic. It has been reported that less than 10% of the research papers fulfill the status of classic articles.^16^ In larger research fields, articles with more than 400 citations are considered classics. However, the classic citation varies for each field.^17^ In the present study, 34 articles had more than 100 citations and could be categorized as classic. This higher proportion is not surprising, since many of these publications pertain to the newer advances in the field, increasing in research and publications, and the tendency for authors to cite preferentially articles with higher levels of evidence.^18^

Time since publication is an essential factor that impacts the citation metrics of an article.^8^ Older articles receive more citations than recently published ones, due to the advantage of time and the snowball effect of subsequent related articles referring to older and primary articles.^8^ ^,^ ^19^ Correlating with other studies,^19^ ^,^ ^20^ more cited articles were published after 2010, highlighting the scientific expansion in Orthodontics focused on clinical trials and evidence-based practice. It is important to note that in the Cochrane Database of Systematic Reviews (IF: 8.4), an internationally recognized evidence-based Medicine journal, a handful of articles (n=5) were published. This might be due to the scarcity of clinical trials in Orthodontics to conduct well-designed MAs. 

The IF of a journal is another factor influencing the citation metrics, and accounts for nearly 59% of the citation discrepancy.^21^ In this study, top-cited SR and MA citations ranged between 45 and 344, and were published in high impact orthodontic journals. The IF of the journals included in this study ranged from 1.9 to 10.5, with a median of 3. The relationship between IF and the number of citations is bidirectional and mutually beneficial. High IF indicates high repute, visibility, and readership for the journal among peers.^22^ This motivates the researchers to select these journals to publish their high-quality research. By virtue of quality, these publications inherently have a high potential for citations and boost the IF further over time.

Countries with better economic rankings are likely to publish the most impactful papers, which may be related to the availability and allocation of resources necessary to undertake such studies.^23^ In agreement, 21 out of 100 articles were funded in this study, most of which belonged to developed nations (Supplementary Table). This study showed that 65 of 100 articles were from the top 10 countries in world economic rankings, based on GDP in 2023.^24^ Concordant with similar studies,^4^ ^,^ ^10^ ^,^ ^25^ the majority of corresponding authors were from Europe (n=62), with the United Kingdom (n=15) and Italy (n=14) being top contributors. 

Another noteworthy observation was the number of authors involved with these publications. These top 100 articles were co-authored by 351 authors affiliated with 104 universities. The number of authors per paper varied from 1 to 10, with more than five authors in 58 publications. Further, 53 of 100 were international collaboration or multi-university research papers. These reiterate that collaboration is vital in elevating the impact of articles, and collaborative papers are expected to be more cited.^26^

Keywords of scientific literature define the research field or topic, and enhance the visibility among peer researchers.^27^ It is also essential to be in words rather than phrases or sentences. Therefore, it serves as a code for locating the required article.^17^ ^,^ ^27^ It is no surprise that the most often used term was Orthodontics, followed by Systematic Review and Meta-analysis, given that this study focuses on SR and MA of orthodontic literature. Interestingly, considering that the maximum number of articles were based on bone anchorage and biomechanics, very few keywords were related to it. Clear Aligners (n=6) and Invisalign (n=4), related to the Aligner domain, were most commonly used. Most journals require keywords while submitting the manuscript, but it was unusual that many articles were without keywords. Some of the high-impact journals in the field of Orthodontics, like AJODO and the European Journal of Orthodontics (EJO), did not contain keywords (Supplementary Table). On the other hand, AJODO and EJO recorded the highest citation and maximum number of articles (n=56) among the top 100 cited articles.

## LIMITATIONS

Using only Clarivate Analytics Web of Science’s (WoS) Science Citation Index (SCI) to identify the top-cited articles is a limitation. WoS gathers information from academic journals, books, book series, reports, and conferences. It provides access to current information and historical data from 1900 onwards for more than 8850 of the world’s most renowned academic journals in 150 scientific fields. Other options include the Scopus database, which tracks citations from 1996, which is a downside for considering citation analysis. Similarly, Google Scholar includes books, conference papers, theses, dissertations, unpublished data, and reports, influencing the citation count. However, WoS remains the most significant and widely utilized source database for bibliometric and citation analysis across all academic disciplines.^28^ ^-^ ^30^ The current study may have missed articles published in non-indexed and non-English journals. The number of citations decides the impact and quality of an article; unfortunately, it could be time-dependent. Another shortcoming is the potential source of error in such bibliometric studies resulting from ‘self-citation’^31^ and ‘journal bias’. The former indicates the authors’ tendency to cite their publications, to improve their credentials and journal IF. Journal bias refers to the inclination of the authors to cite papers from the same journal targeted to publish their research.^32^ ^,^ ^33^ Finally, the author’s actual affiliations were only considered if multiple institutions were present.

## CONCLUSION

This descriptive bibliometrics analysis provides scientific evidence mapping of orthodontic literature. The 100 top-cited SR and MA in orthodontics were published from 1996 to 2021, with high-impact orthodontic journals (AJODO, EJO, and Angle Orthodontist) contributing the most. Thirty-four of them had already grossed a hundred citations or more. OTM/biomechanics and Bone anchorage were the trending domains. The articles were often multi-authored and involved collaborative work from different universities. Europe was the most productive in terms of authors and institutions. The findings may be of interest and useful to all prospective authors and synthetic research studies in Orthodontics.

## Supplementary Table: Descriptive data of top 100 cited systematic review and meta-analysis in orthodontics.

[Fig f1app]
